# Automating the bottleneck: a scalable deep learning pipeline for phytolith detection, imaging, and curation across single-cell and multi-cell morphotypes

**DOI:** 10.3389/fpls.2026.1926651

**Published:** 2026-09-25

**Authors:** Melanie Pugliese, Lachlan Kyle-Robinson, Hazim Elgamal, Xintong Gao, Nicolas Iovu, Kevaan Buch, Iban Berganzo-Besga, Monica N. Ramsey

**Affiliations:** 1 University of Toronto, Toronto, Canada Ramsey Lab for Environmental Archaeology, Mississauga, ON, Canada; 2 Barcelona Supercomputing Center - Centro Nacional de Supercomputacion, Barcelona, Spain

**Keywords:** automated workflow, microscopy - brightfield, paleoethnobotany, phytoliths, plant identification

## Abstract

Deep learning offers powerful tools for automating phytolith classification. Although foundational models have enabled image classification with little or no task specific training, specialized domains such as phytolith images still require a labor-intensive process to acquire labelled training data. This article presents a novel, scalable imaging workflow that addresses this bottleneck by integrating low-magnification slide scanning with artificial intelligence assisted phytolith detection and targeted high-magnification 3D Z-stack capture optimized for both multi-cell and single-cell phytoliths. Automated pre-processing enables imaging of 16 slides per week generating 100’s of curated phytolith training images per slide. Developed in collaboration with Nikon Imaging Specialists and validated on 34 comparative grass and rush taxa, the system combines expert-guided sampling with customized automated acquisition routines to capture, segment, tile and curate images for deep learning input. Key workflow optimizations, including hardware upgrades, asynchronous file conversion, and iterative refinement of the artificial intelligence detection model, achieved a 350% improvement in extended depth of field rendering speed and a 55,450% improvement in TIFF export speed, enabling sustained high-throughput acquisition without performance degradation. The workflow was designed to optimize imaging of mixed multi-cell and single-cell phytolith assemblages and is currently generating training data for DL models targeting wild versus domesticated wheat discrimination in addition to grass species identification from multi-cell husk phytolith morphology. Beyond phytoliths, the pipeline is modular, replicable across microscopy platforms, and readily adaptable to other plant microfossils and microstructures, including pollen, starch grains and other silica bodies, through targeted artificial intelligence retraining, making large-scale machine learning applications in plant science, paleoethnobotany and related fields more accessible and reproducible.

## 1 Introduction

Tracking changes in ancient plant use at a meaningful scale requires robust, taxonomically diverse datasets drawn from the full range of plants cultivated, processed, and consumed by ancient humans worldwide. One source of such data is phytoliths, microscopic silica bodies formed within and between plant cells as absorbed silica is deposited throughout plant tissues, producing both single-cell and multi-cell morphotypes (Piperno, 2006). These structures can be diagnostic at multiple taxonomic levels, enabling plant type identification through morphological criteria (Jenkins et al., 2011; Nawaz et al., 2019). At the family and subfamily levels, single-cell morphotypes can indicate taxonomic affiliation, plant part, and broad environmental conditions such as wetland, temperate, or semi-arid habitats (Rosen, 2013). Providing finer resolution, multi-cell forms can be diagnostic to the genus level, making them particularly valuable for identifying key cereal grasses such as wheat and barley in the archaeological record (Rosen, 1992; Ball et al., 1996, 1999).

Phytolith analysis has been successfully applied across diverse geographical and cultural contexts owing to the taphonomic robustness, and widespread preservation of these structures (Pearsall, 2015). Studies have reconstructed artifact use, foodways, cuisine, mortuary practices, diet and dietary change over time (Abramiuk et al., 2011; Cagnato, 2018; Durán et al., 2021; Herrera Parra et al., 2026; Morell-Hart et al., 2014, 2021; Pugliese, 2023; Berganzo-Besga et al., 2021), as well as ethnoecology, and agricultural practices (Ramsey, 2023; Solís-Castillo et al., 2015; Stivrins et al., 2025; Watson, 2022; Kyle-Robinson et al., 2025, 2026). Geographically, applications span Mesoamerican assemblages rich in grass, root crop, flowering species and palm morphotypes that have transformed knowledge of ancient landscape management and cuisine (Herrera Parra et al., 2026; Morell-Hart et al., 2021), Near Eastern deposits where wetland and cereal taxa illuminate early sedentary lifeways and agriculture origins (Ramsey, 2023; Ramsey et al., 2017; Ramsey and Rosen, 2016; Rosen, 2013), and Southeast Asian and African contexts where phytolith analysis is reshaping our understanding of early domestication and land use (Carson et al., 2025; Kinyanjui et al., 2026; Zhang et al., 2025). Beyond paleoethnobotany, phytoliths have also proven valuable for reconstructing paleoenvironments (Stiles et al., 2025) and elucidating plant evolution through phylogenetic analysis (Brightly et al., 2024; Hošková et al., 2022). Taken together, these studies confirm that phytoliths represent one of the most durable and geographically versatile proxies available to paleoethnobotanists and associated researchers alike (Pearsall, 2015; Piperno, 1998).

Interpreting phytolith data, however, requires contending with taphonomic and analytical challenges rooted in how silica is deposited across plant taxa. Monocotyledons including grasses and rushes (e.g., corn, wheat or millets) deposit silica within their cells, producing abundant and morphologically distinctive phytoliths that are more easily identifiable in archaeological assemblages (Pearsall, 2015). In contrast, Dicotyledons (flowering plants including roots and tubers) deposit silica between cells, yielding fewer and less diagnostic forms (Pearsall, 2015; Piperno, 1998). Beyond this structural difference, phytolith production varies significantly across taxa, meaning some plants are consistently underrepresented regardless of preservation conditions. Together, these biological factors render phytolith assemblages inherently biased, reflecting production and preservation processes as much as actual patterns of past plant use (Jenkins and Rosen, 2007; Neumann et al., 2017). Compounding these taphonomic constraints, current microbotanical research is further limited by the slow speed of sample processing and identification, which often restricts studies to small sample sizes spanning broad regions and time periods. Moreover, traditional phytolith analysis is reliant on individual expertise and thus prone to interpretative bias, limiting both taxonomic resolution and broad application (Florin and Ramsey, 2026; Out et al., 2025; Ramsey et al., 2026). Collectively, these constraints risk producing simplified narratives that may obscure the true complexity of past subsistence patterns.

Recent breakthroughs in the use of artificial intelligence (AI) for applications in archaeology showcases the utility of this tool, increasing accuracy and efficiency across a broad range of applications (Anagün et al., 2025; Argyrou et al., 2023; Bickler, 2021; Fetaya et al., 2020; Fiorucci et al., 2020; Olsson et al., 2021). Within the field of paleoethnobotany, several research groups are transforming phytolith identification through the application of AI by offering a more consistent, scalable and objective alternative to manual classification. Morphometric analysis, involving quantitative measurements of phytolith shape and size, has been shown to enhance taxonomic resolution, particularly for morphologically similar types (Ball et al., 2016). Building on this foundation, Cai and Ge (2017) created a machine learning (ML) pipeline that combined morphometrics and model training, reaching 87.7 percent accuracy across more than 1,000 samples. Díez-Pastor et al. (2020, 2024) refined these methods through feature testing and classifier stacking, showing that shape and appearance descriptors produced the highest accuracies when paired with convolutional neural networks (LeCun et al., 1998; Krizhevsky et al., 2012) and Vision Transformers (Dosovitskiy et al., 2021). Evett and Cuthrell (2016) proposed an early framework for automated phytolith analysis centered on segmentation, feature extraction, and searchable databases, emphasizing reproducibility and standardization. Gallaher et al. (2020) expanded this by using three-dimensional morphometrics, achieving up to 90 percent accuracy for short-cell grass phytoliths. Brightly et al. (2024) applied Random Forest models to palm phytoliths across 97 modern species spanning five subfamilies, obtaining 59 percent accuracy at the clade level, with petasoid echinate forms performing best. Berganzo-Besga et al. (2022) developed the first DL workflow for multi-cell phytoliths using a pretrained VGG19 model with image augmentations, reaching 93.68 percent genus level accuracy and setting the stage for interpretability research with Guided Grad CAM (Selvaraju et al., 2017; Berganzo-Besga et al., 2025).

Together, these studies point to a growing consensus: successful automated phytolith classification relies on high-quality imaging, thoughtfully engineered features, and large diverse training datasets. While current models demonstrate strong performance, particularly within well-defined taxonomic groups, broader phylogenetic generalization and effective handling of the variability inherent in archaeological phytolith samples both represent key frontiers for future advancement in the field.

Despite these advances, a major bottleneck in applying Deep learning (DL) to phytolith classification is the limited availability of high-quality, labeled training datasets. Traditional phytolith workflows are slow, labor-intensive, and difficult to scale often requiring manual selection and photography. This constrains both the size and taxonomic diversity of available training datasets, reducing classification accuracy and limiting generalizability across taxa and archaeological contexts. Beyond dataset size, the composition of training data is equally critical. For example, unequal class balancing, where some classes are represented by far more examples than others, cause models to optimize for majority classes during training, systematically underperforming on rare but often diagnostically important morphotypes, inflating performance metrics and overstating practical utility (Buda et al., 2018) (see **Table 1**). This is a common and frequently unaddressed problem across existing phytolith AI studies, with class imbalance either unresolved or unreported in the majority of cases. The workflow positioned here provides researchers the tools needed to produce and sustain a growing collection of high-quality phytolith training images to mitigate this limiting factor.

**Table 1:** Training dataset characteristics across AI-based phytolith classification studies.

Study	Total labeled images	Per-class range	Balanced dataset*
Cai and Ge (2017)	1,063 *23 taxa*	~46 avg *per-class breakdown not reported*	Unclear — per-class distribution not reported
Díez-Pastor et al. (2020)	429 *8 morphotype classes*	~54 avg *per-class breakdown not reported*	Not reported
Díez-Pastor et al. (2024)	429 *same database reused from 2020; 8 morphotype classes*	~54 avg *same as 2020; not reported per class*	Not reported
Gallaher et al. (2020)	1,093 3D models *selected from 21,632 observed; 48 genera, 21 subtribes*	~10–30 per class *severe class imbalance explicitly acknowledged by authors*	No — imbalanced; authors note bias toward larger groups
Brightly et al. (2024)	2,534 *9 palm clades + 4 outgroup families*	~8–204 per group *imbalanced; model bias explicitly noted by authors*	No — imbalanced; bias toward two largest groups documented
Berganzo-Besga et al. (2022)	1,470 (dataset 1) and 585 (dataset 2) *3 genera, 5 classes; +58,065 augmented training images*	~490 per genus (dataset 1) *~195 per class (dataset 2); deliberately balanced*	Yes — equal class representation (33.33% per genus; 20% per class in 5-class model)
Ramón et al. (2026)	12,518 with 2D + 3D representations *24 morphotypes*	17–532 per morphotype (30-fold range) *Note: authors state minimum 20 labelled observations per class for inclusion, but Achene Body has only 17 in the reported train+test dataset (Table S.B4)*	No — imbalanced

*Blue indicates adequate dataset balancing. Yellow indicates partial or unclear reporting. Red indicates absence of balancing or inadequate per-class representation..

This paper addresses the methodological gap between microbotanical sampling and AI application. It introduces an integrated imaging pipeline that automates key steps in the capture and pre-processing stages of training data creation and ultimately may be applied to archaeological data. The goal is to support generation of a balanced and taxonomically diverse training dataset that minimizes manual labor without sacrificing image quality, ensuring the feasibility of scaling ML applications across diverse taxa and increasing taxonomic resolution. Specifically, the workflow aims to:

1. Automate image acquisition
2. Streamline image processing using automated tiling and segmentation techniques
3. Enable consistent and reproducible data generation across a range of plant taxa and phytolith types (i.e., large-multi-cell, small-multi-cell and single-cell)
4. Support future DL model training by producing a large volume of standardized, high-quality images to address current limitations in phytolith classification training datasets

While developed for phytolith analysis, the pipeline is designed to be modular and conceptually platform agnostic, with potential applicability to other plant microstructures, where high throughput image acquisition and AI-assisted detection could similarly accelerate training data generation.

## 2 Materials and equipment

### 2.1 Plant material

Comparative plant material for phytolith analysis was sourced from multiple collections to ensure taxonomic breadth and regional relevance. Samples were drawn from Dr. Monica N. Ramsey’s Near East collections housed at the Ramsey Laboratory for Environmental Archaeology (RLEA) at UTM, which pull from the UCL Hillman collections (Drs. Monica N. Ramsey and Heather Miller both subsampled this collection at different times) and the Mt. Scopus Collection. The Hillman collection is focused specifically on Near Eastern grass husks, including 46 wild and domestic grass specimens while the Mt. Scopus collection focused primarily on wild plant taxa, including 148 economically important specimens from across the Levant. A total of 33 grass taxa from the Poaceae and one rush taxon from the Juncaceae family were processed, spanning the genera *Triticum*, *Hordeum*, *Avena*, *Aegilops*, *Bromus*, *Festuca*, *Stipa, Lolium* and *Juncus*.

### 2.2 Reagents and consumables

The following reagents and consumables were used for sample preparation and slide mounting: distilled water (rinse), porcelain crucibles, sterile metal spatulas, labelled glass vials (ash storage), standard glass microscope slides, and Entellan mounting medium (Sigma-Aldrich) for slide preparation.

### 2.3 Equipment and software

All plant material was ashed using a Paragon High Fire Kiln (SENTRY Xpress 4.0) and image acquisition was performed using a Nikon Eclipse Ni-E microscope equipped with a Nikon Digital Sight 10 Camera. Stage movement, focus, Z-stack acquisition, extended depth of field (EDF) rendering, automated job execution, and AI-based region of interest detection were all controlled through the NIKON NIS-Elements software (AR 6.02.03). A 10× plan apochromat objective was used for low-magnification full-slide overview scanning, and a 40× plan apochromat objective was used for high-magnification Z-stack acquisition. All imaging was performed under brightfield transmitted light illumination.

The original imaging workstation was an HP Z4 G5 equipped with an Intel Xeon w3–2425 processor, an NVIDIA RTX A4000 GPU, 128 GB DDR5 RAM (4400 MHz), and 11TB of HDD storage. Workflow development and iterative optimization (described below) revealed this configuration to be a significant throughput bottleneck. A 4TB NVMe SSD (Samsung 990 Pro) was added to the original workstation to address storage-related I/O constraints. The system was subsequently upgraded to a custom-built workstation equipped with an AMD Ryzen 9 9950X3D processor, an NVIDIA RTX 4070 GPU, 128GB of DDR5 RAM (6000 MHz), and 5TB of PCIe Gen5 SSD storage. GPU selection was constrained by compatibility requirements of Nikon NIS-Elements software, which limited the system to a mid-range RTX-class card. The NIS-Elements job database was relocated to SSD storage as part of this upgrade, which proved essential for sustained high-throughput acquisition.

The automated workflow relies on a suite of custom software tools and shared cloud infrastructure. All custom desktop applications were coded in python and web-based apps were coded in React and hosted on Vercel. A custom desktop application handles asynchronous TIFF-to-JPG conversion and upload to centralized cloud storage. Two separate custom sorting applications, one desktop and web-based, and one web-based only, support tile annotation and bounding box annotation respectively, with file operations executed asynchronously and in batches. A computer application runs the segmentation pipeline originally developed by Berganzo-Besga et al. (2022) for multi-cell phytolith processing.

## 3 Methods

### 3.1 Plant processing

Each plant specimen was subdivided into distinct anatomical components including, leaves, stems, roots, inflorescence and seeds, which were processed independently to capture tissue-specific phytolith production and morphological variation. Since phytolith morphotypes can vary significantly within a single plant, separating anatomical parts allows for the generation of a more representative training dataset and reduces bias towards any single tissue or individual specific morphotype. To further address intraspecific variability, multiple individual plants per species were sampled when available, to avoid over-representation of a single phenotype or growth condition. This strategy reduces the risk of training the model on a potentially unrepresentative morphotype and improves generalization performance during validation and external testing.

### 3.2 Sample preparation and slide mounting

All plant material was subjected to controlled dry ashing prior to slide preparation. Plant samples were first rinsed with distilled water. Dried anatomical plant parts were then placed in porcelain crucibles and combusted at 555 °C for 3 hours 33 minutes in the kiln to remove organic matter while preserving silica phytolith structures. Following combustion, crucibles were left to cool overnight. Complete ashing was confirmed by the uniform grey appearance of the residue. Any remaining black material indicated incomplete combustion and required an additional firing cycle. The resulting ash was gently homogenized using sterile metal spatulas to reduce clumping and promote even distribution of phytoliths. The homogenized ash was transferred into labelled glass vials for storage.

For each sample, a small quantity (0.8-1mg) of the ash was transferred onto standard glass microscope slides and mounted using Entellan mounting medium. Slides were left to dry horizontally for a minimum of two weeks before imaging. The quality of slide preparation at this stage directly influences downstream image quality and phytolith density: thorough plant part sorting prior to ashing and careful, even distribution of ash during mounting produces slides with higher phytolith density and reduced background debris, which in turn increases conversion rates (ratio of high-quality images) during the curation phase (see Results).

### 3.3 Workflow overview

The complete workflow proceeds in three broad phases: (1) slide preparation and low magnification full-slide scanning; (2) AI-assisted phytolith detection and automated high-magnification Z-stack acquisition; and (3) image export, conversion, segmentation (multi-cells), and supervised annotation. In the automated configuration described here, a single operator command initiates the complete imaging sequence, with the system proceeding from full-slide scan through AI detection to high magnification acquisition without further manual input.

#### 3.3.1 Original manual imaging workflow

The original training data acquisition workflow involved capturing full-slide scans of ashed comparative samples at 10× magnification, providing a comprehensive overview while minimizing file size. These scans were reviewed in Nikon NIS-Elements and points of interest (POI) containing diagnostic phytoliths were manually identified and annotated using the ND Acquisition Wizard with typically, 150–300 coordinates selected per slide (focused mainly on multi-cell imaging). High-magnification imaging was then performed at each coordinate using automated Z-stacks with continuous in range autofocus. Each Z-stack comprised approximately 10 focal layers (max ~15µm) for single-cell and small multi-cell phytoliths and up to 35 layers (~68µm) for large multi-cell phytoliths (**Figure 1**). Stacks were rendered using EDF processing and exported as TIFF files, which were organized into labeled directories, and uploaded to a cloud-based storage for integration into the DL training pipeline. Each week, four microscope slides were imaged, representing either distinct plant species, genera, or multiple individuals of the same taxon to capture intraspecific variability. Imaging proceeded until either the entire slide had been scanned or approximately 300 individual phytolith images had been acquired per slide. Manual point selection and imaging under this workflow required a minimum of 3–4 hours of continuous operator time per slide. This hybrid workflow, developed in consultation with Nikon Imaging Specialists, reduced manual microscope manipulation while preserving expert oversight, but remained limited in throughput and scalability.

**Figure 1:**
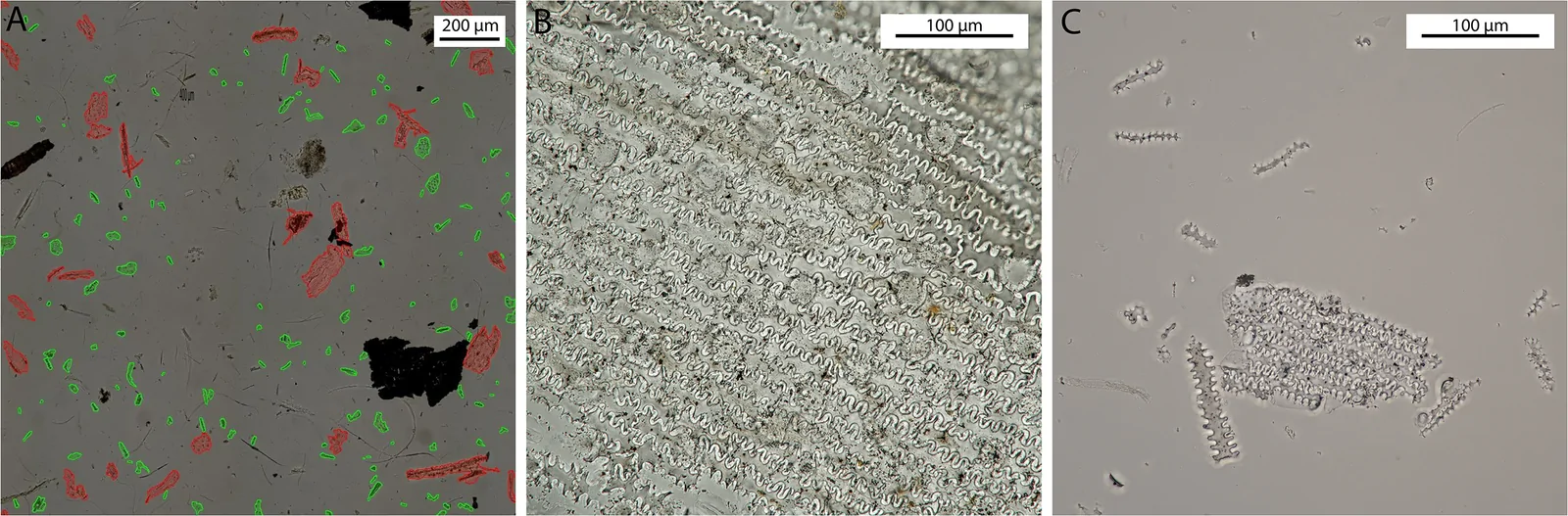
Phytolith class separation. **(A)** Automated point of interest (POI) selection of the two phytolith classes. Large multi-cell phytoliths are masked in red. Small multi-cell phytoliths and single cell phytoliths are masked in green **(B)** Large multi-cell phytolith **(C)** Small multi-cell phytolith and single cell phytoliths.

#### 3.3.2 Design constraints and iterative refinement

Development of the training data pipeline revealed a series of physical, optical and computational constraints that shaped the final automated workflow. Early trials explored Z-stack 3-D full-slide scanning at 40× magnification to maximize morphological detail and eliminate the need for a separate 10× overview scan. Although these scans produced exceptionally rich imagery, individual file sizes exceeded 2 TB, rendering storage, transfer, and downstream processing impractical. This prompted evaluation of alternative file formats and whether EDF compositing was necessary during overview slide scan acquisition; however, these alternatives redistributed rather than resolved the computational burden, which was ultimately constrained by memory bandwidth and storage throughput.

Subsequent experiments evaluated full-slide scanning at 20× magnification and assessed whether Z-stacks were required for overview imaging. Four full-slide scans at 10× already required approximately six hours and generated files on the order of 8.5 GB. Testing demonstrated that 10× overview scans with approximately 11 focal layers (2 µm steps) were sufficient to capture the depth range in which phytoliths occur on the slide, and that higher stack counts did not yield additional diagnostic information.

The optical requirements of large and small phytoliths also proved incompatible within a single acquisition routine. Large multi-cell phytoliths require approximately 25–35 focal layers (48 - 68µm) to capture their full 3-D depth, whereas small multi-cell forms require 10–15 layers (max ~20µm) and small single-cell forms require only 10-15 layers (0.5 µm steps). Imaging single-cell and both small and large multi-cell phytoliths within a unified protocol either truncated large multi-cell phytoliths or wasted substantial acquisition time on smaller multi-cell and single-cell phytoliths, due to the inclusion of unnecessary focal layers, creating poor quality images and unnecessarily large files. This ultimately motivated their separation into distinct imaging classes, formalized in the automated segmentation workflow.

#### 3.3.3 Nikon AI training and phytolith annotation

A 40× slide scan file was imported into Nikon NIS-Elements for AI training and annotation. The training dataset consisted of a full slide scan processed using EDF rendering but retained in its native tiled format rather than stitched into a single composite image, preserving hundreds of discrete images for annotation. This file contained approximately 250 images of multi-cell husk phytoliths, each containing a mixture of large multi-cell and smaller single-cell phytoliths. The exact number of individually annotated objects was not recorded. Manual segmentation was performed using the *Binary Manual Segmentation tool*, which was employed to generate image masks for the two size classes: large multi-cell phytoliths (25-35 focal layers), and small multi-cell and single-cell phytoliths (10–15 focal layers). The annotated dataset was used to train the Nikon AI detection model for 1,000 iterations, reaching a final training loss of 0.20021 Model architecture, augmentation strategy, and hyperparameters are proprietary to the NIS-Elements GA3 environment and are not exposed to the user. Training iteration count and training loss were the only metrics available for monitoring model convergence. The trained model was subsequently deployed to identify candidate phytoliths based on morphology and size across new 10× slide scans, generating coordinates for downstream 40× acquisition. This cross-magnification approach was recommended by Nikon Imaging Specialists based on their experience with the NIS-Elements GA3 platform. The underlying technical mechanism enabling detection across magnification is proprietary and was not disclosed to the authors.

#### 3.3.4 Automated slide scanning and high-magnification imaging

The automated imaging and acquisition workflow (**Figure 2**) begins with low magnification acquisition at 10×. A fast autofocus routine is performed within a 200 µm range, followed by definition of a symmetric Z-stack. Standard Z-stack parameters were set to a 20 µm range with 11 steps at 2 µm intervals, although these parameters were adjusted as needed based on phytolith size, density, and slide topography. Z-stack parameters were established during the manual workflow optimization phase. Acquisition range and step interval were tested across a series of slides and the final parameters were selected on the basis of producing the highest image clarity and morphological definition in EDF composites for Entellan mounted material. These parameters have been applied consistently across all subsequent automated acquisition sessions. Imaging points for low-magnification capture (10×) were generated using a *GeneratedPoints* operation executed within a Well Loop structure (configurable working area boundaries, area restrictions and scan direction) for each slide compartment within the microscope stage. The resulting 10× slide scan provides the input dataset for AI-based region of interest detection (two size classes: large multi-cell; small multi-cell and single-cell phytoliths).

**Figure 2:**
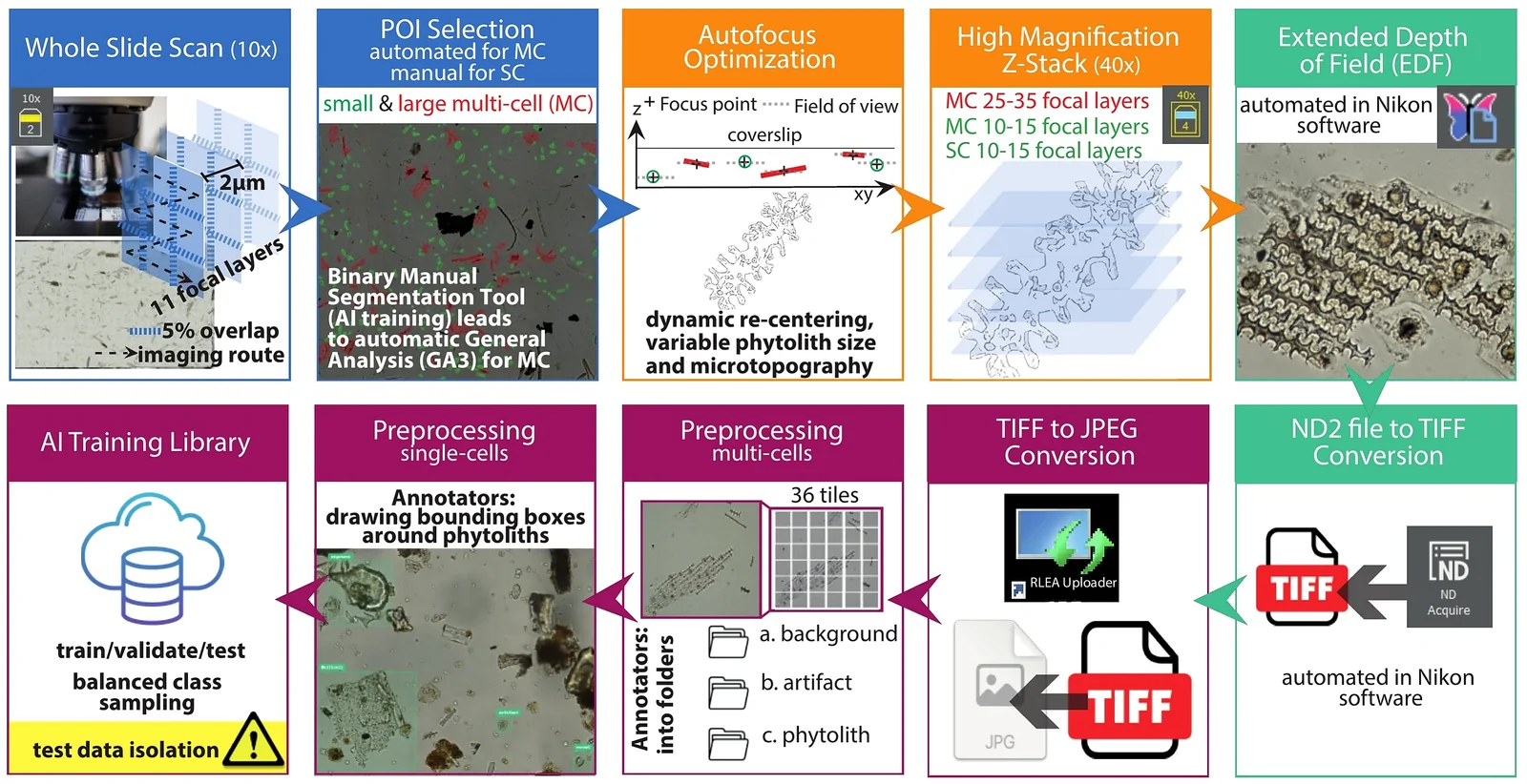
Data pipeline architecture for high-throughput phytolith image acquisition, processing, and annotation. The workflow is organized into four integrated components distinguished by color. Automation was executed using the JOB function in NIS-Elements, which allows programmable control of multi-step imaging procedures. 1) BLUE (Automated whole slide acquisition and POI detection): Whole-slide scans are acquired at 10× magnification, followed by automated point-of-interest (POI) selection targeting 2 classes: large multi-cells; and small multi-cells and single-cell phytoliths. A binary manual segmentation tool is used to guide automated general analysis for refined POI detection. 2) ORANGE (High-magnification imaging and in-microscope processing): Selected POIs are imaged at 40× using Z-stack acquisition with variable focal depths tailored to large multi-cell, and small multi-cell, and single-cell structures. Acquisition is further optimized through continuous autofocus routines, dynamic re-centering, and adaptive positioning to account for variability in phytolith size and morphotype. 3) GREEN (NIKON Processing of 3D Images) Extended depth of field (EDF) rendering is applied to produce fully in-focus images, which are then exported as TIFF files. 4) PURPLE (External processing, annotation, and dataset construction): TIFF images are automatically converted to JPG format within a designated output directory and uploaded to cloud storage. Preprocessing is initiated through a custom application, where users select input files from the cloud and define output directories. Multi-cell images are subdivided into 36 standardized tiles and annotated into background, artifact, or phytolith classes. Full-resolution images are additionally loaded into a separate interface for single-cell annotation using bounding boxes. The final output is a curated, balanced training dataset suitable for downstream machine learning applications. Balance is achieved by requiring each taxon to reach 500 curated training images before it is admitted to model training. Taxa below this level remain under active imaging rather than being included as finalized classes. The AI Training Library panel shown in the diagram represents the model train/validate/test stage that follows dataset curation. This stage is under active development and is shown here to indicate the pipeline’s intended downstream use for prospective adopters.

Following completion of the 10× scan, a *General Analysis* (GA3) operation applies the trained AI model to identify large multi-cell and small multi-cell/single-cell phytoliths, generating coordinates for high-magnification acquisition (**Figure 3**). The JOB then generates 40× imaging points corresponding to these regions. At each coordinate, a fast autofocus routine is performed prior to image acquisition, and each region is captured as a 40× Z-stack over a specified range, for example multi-cell’s require a minimum range of 48µm, with 25 steps at 2µm intervals, using continuous in-range autofocus to maintain optimal focus. These acquisition parameters (Z-stack range, step interval, EDF rendering) are identical to those in the original manual workflow (Section 3.3.1), established during the manual optimization phase described above. The automated workflow therefore alters point selection and capture triggering but not the underlying image formation parameters. Z-stacks are then processed using EDF rendering to produce high resolution composite images suitable for downstream segmentation and DL applications. A custom macro converts output files to TIFF format to standardize file output for subsequent processing. Prior to EDF rendering and TIFF export, the *Keep Selected Frames* function is enabled within NIS-Elements, reducing each output file to a self-contained unit and ensuring file sizes remain manageable for sustained batch processing.

**Figure 3:**
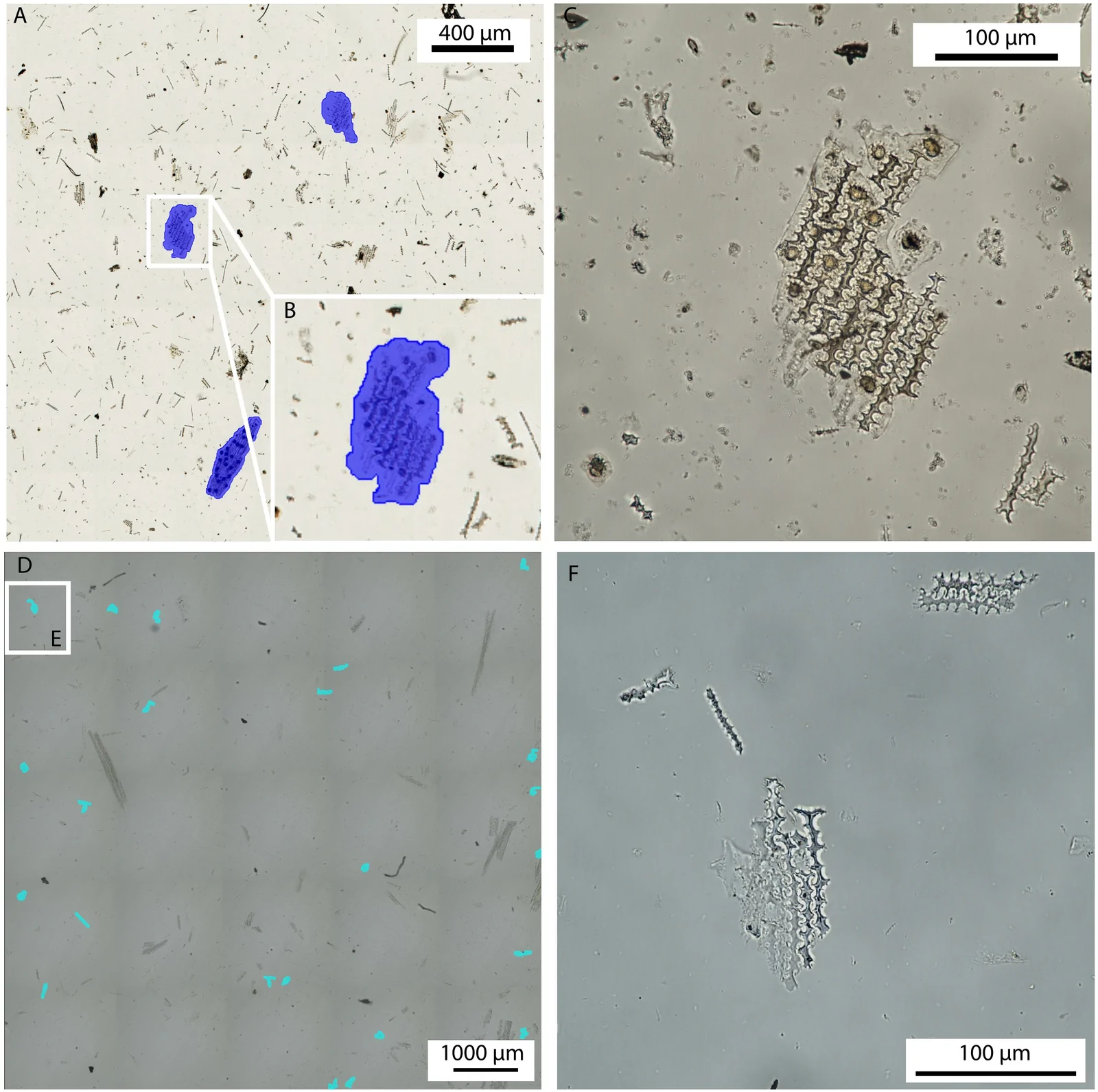
Multi-cell POI Selection From 10× slide scan of Grass Husk. Image displays POI selection **(A)** POI selection from GA3 following full slide scan of large multi-cells **(B)** Masked identified phytolith X and Y coordinates are cued for high magnification Z-stack imaging **(C)** EDF rendered final 3-D TIFF image **(D)** POI selection from GA3 following full slide scan of small-multi cells and single cells. Filter area greater than 10,000 µm², smaller than 500,000 µm² **(E)** Masked identified phytolith X and Y coordinates are cued for high magnification Z-stack imaging **(F)** EDF rendered final 3-D TIFF image.

#### 3.3.5 Automated workflow optimization

The finalized JOB reflects a series of improvements aimed at making image acquisition more efficient, keeping data organized, and ensuring compatibility with the downstream DL workflow. Rather than applying EDF rendering as a separate step afterwards, it was built directly into the JOB. These standardized outputs eliminated the need for manual oversight. To make this work correctly, the step responsible for EDF processing was moved to run on the complete Z-stack file, which contains all stacks from every imaged point, rather than as a separate process that needs to be initialized manually.

File organization was also cleaned up. Earlier versions saved each image slice and imaging point as its own file, which fragmented the data and created unnecessary load when reading and writing files. Disabling the *Split Storage per Point* function on NIS element software and moving EDF processing outside the automated point loop (40× capture, fast autofocus, and Z-stack loop) means the workflow now produces a single, consolidated output for each field of view instead.

Detection parameters were further refined after the initial Nikon AI detection model was found to be occasionally selecting very small phytolith fragments resulting in imaging of the same location multiple times. An area-based object filtering step (**Figure 4**) was integrated by linking size filtering parameters to the high-magnification GA3 operation, enabling exclusion of phytoliths below a defined area threshold. Illumination consistency at 40× brightfield was standardized through automated aperture control stored directly within the optical configurations.

**Figure 4:**
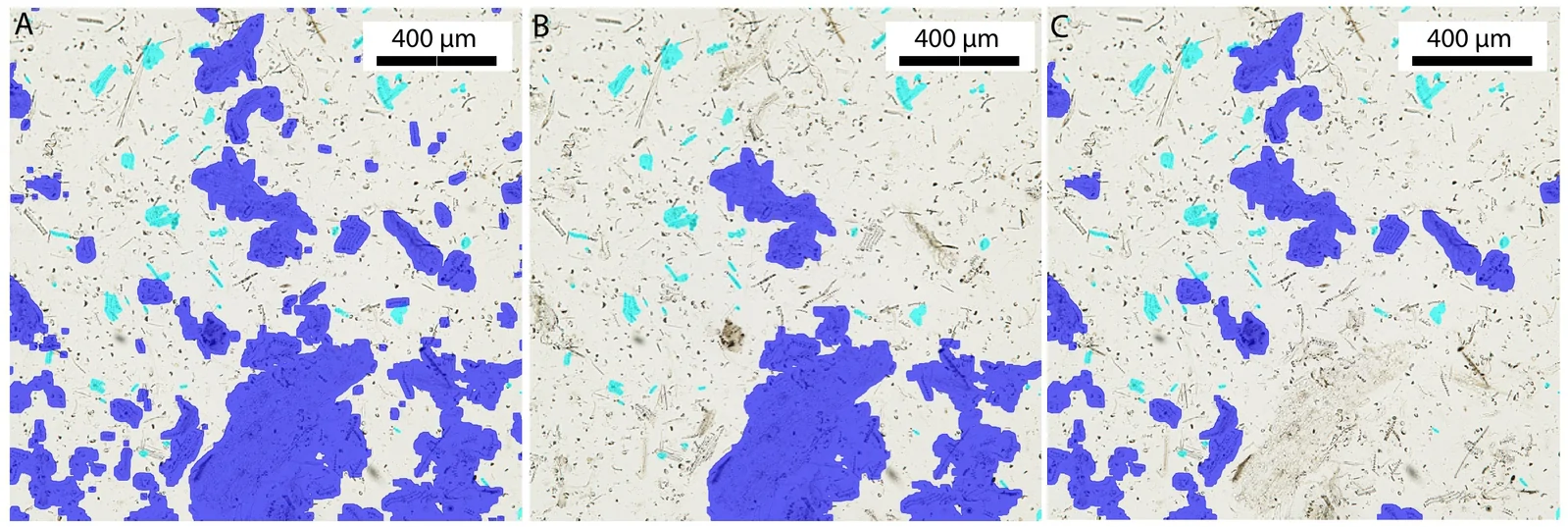
POI filtering. Filtering component added to the POI selection GA3 **(A)** No filtering **(B)** filter area greater than 5000µm² smaller than 100000 µm² **(C)** 500 000 µm² area filtering. Masks shown here represent candidate regions identified by the AI detection and area-based filtering step used to target high-magnification (40×) acquisition. These masks determine image acquisition only and do not constitute final training labels.

#### 3.3.6 Training data processing

Following Nikon acquisition, a custom desktop application converts TIFF outputs to JPG format and uploads them to the centralized Google Drive repository (This can be downloaded from https://github.com/Ramsey-Environmental-Archaeology-Lab/Automating-the-Bottleneck.git). This conversion runs asynchronously. Decoupling image acquisition from data preparation allows NIS-Elements to remain dedicated to microscopy operations while conversion and upload proceed in parallel. The repository provides the code and tools needed to reproduce the downstream processing components of this pipeline (image conversion, segmentation, tiling, sorting, and annotation tools). The underlying image dataset is not included, as these images are actively being used to train ongoing, unpublished deep learning models (see Sect 5.4).

##### 3.3.6.1 Multi-cell segmentation and sorting

Multi-cell phytolith images are subsequently processed using a computer application which employs the segmentation pipeline of Berganzo-Besga et al. (2022), segmenting each JPG into standardized black and white tiles for CNN input (**Figure 5**). A team of trained annotators then reviews processed tiles using the custom sorting application, assigning each tile to one of three categories: phytolith, background, or artifact. From the imaged outputs, approximately 600 images (roughly 21,600 training tiles) are selected and sorted weekly for inclusion in the DL training dataset. This structured workflow enables the rapid expansion of a diverse, consistently labelled image library for a wide range of species, providing the foundation for iterative model improvement.

**Figure 5:**
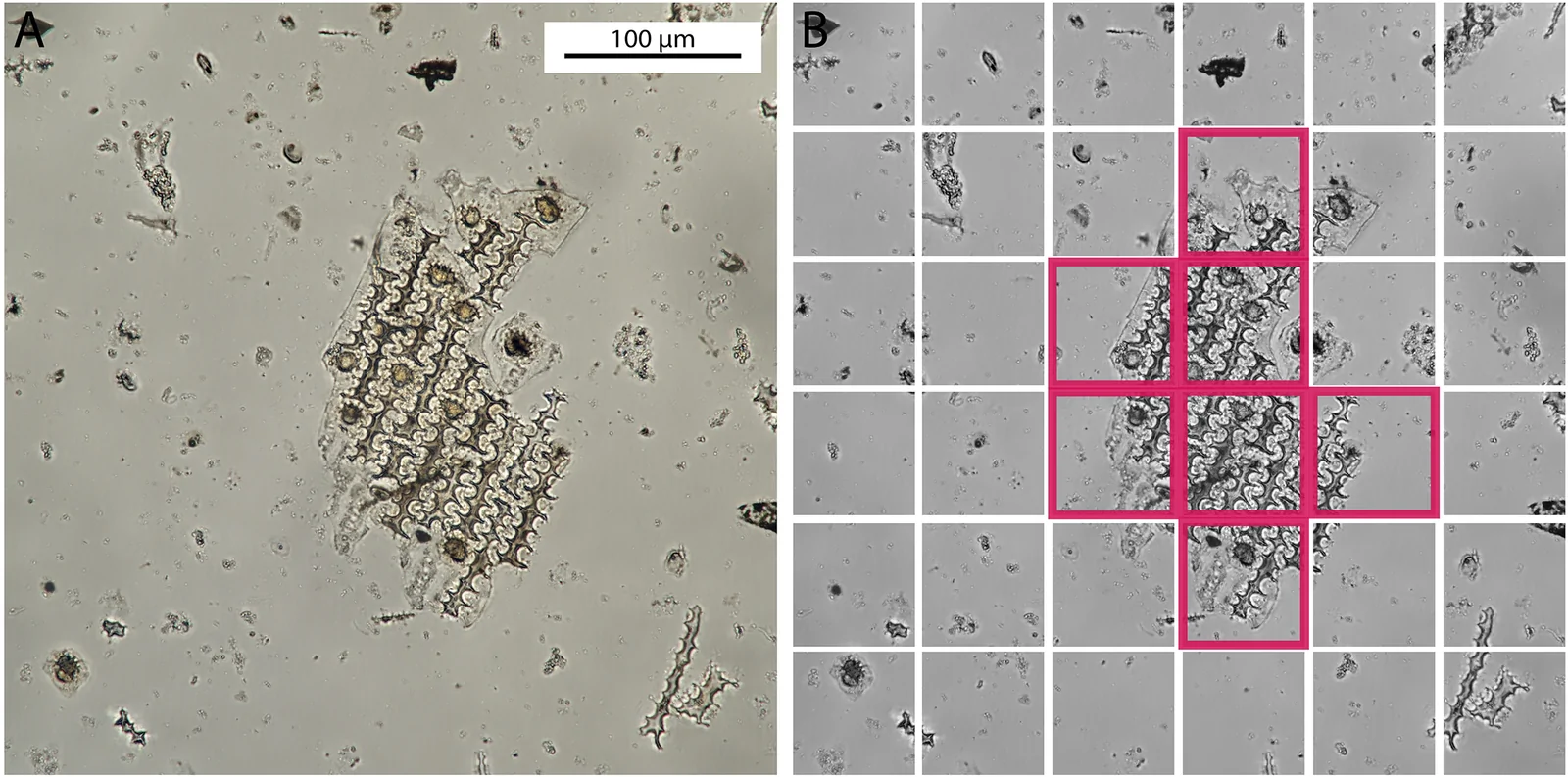
Multi-cell phytolith image processing. **(A)** Full-resolution, color extended depth of field (EDF) composite image of a multi-cell phytolith following Z-stack acquisition and rendering. **(B)** The same image subdivided into the fixed 36-tile (6×6) segmentation grid and converted to greyscale, standardizing image inputs prior to tile-level annotation into background, artifact, or phytolith classes. The pink boxes outline the tiles that would be annotated as phytolith.

##### 3.3.6.2 Single-cell bounding-box annotation tool

A complementary web-based annotation tool was developed to address the specific requirements of single cell phytolith forms, for which the fixed 36 tile grid of the Berganzo-Besga et al. (2022) is not appropriate. The tool loads unprocessed JPG images from either the manual or automated acquisition workflow. Annotators draw bounding boxes around individual phytoliths and artifacts, with support for multiple objects and multiple classes within a single image. Spatial metadata, including position and bounding box dimensions, are saved for each annotation. The tool exports 512 x 512 pixel crops centered on each annotated object, together with the original image. Unlike the tile-based pipeline, which divides images into a fixed spatial grid, this tool is object centered: the annotator determines which phytoliths are included, eliminating truncation at tile boundaries. The tile-based pipeline and the bounding box tool form a complementary pair, with the former optimized for generating training data for multi-cell phytoliths and the latter for single-cell phytolith morphotypes.

## 4 Results

### 4.1 Expected imaging output

Users following this protocol should expect to image approximately 16 slides per week under normal operating conditions, yielding approximately 200–800 phytolith images per slide prior to curation. Unprocessed single-cell JPG images uploaded into cloud storage are loaded into the annotation tool for bounding box annotation. Each image may have upwards of 10 single cells in the image. Each multi-cell image is segmented by the Berganzo-Besga et al. (2022) pipeline, producing approximately 7,200-21,600 tiles per slide before quality filtering. From these, a single trained annotator should be able to sort approximately 1800 tiles (50 full images) in 4–6 hours for inclusion in the DL dataset. This figure reflects sustained throughput under the optimized workflow described here; output during initial setup and AI model training will be lower while annotation teams are trained and acquisition parameters are calibrated for new taxa.

### 4.2 Workflow timing and pause points

**Table 2** summarizes the approximate time requirements for each major step in the automated workflow. Three natural pause points exist at which the protocol can be interrupted and resumed without loss of data or image quality. The first occurs after slide mounting, where prepared slides mounted in Entellan must dry horizontally for a minimum of two weeks and can be stored indefinitely before imaging. The second occurs after TIFF export, where output files can be held in local storage and converted asynchronously without disrupting subsequent imaging sessions. The third occurs when the workflow splits into two distinct processing methodologies; the processing tool for multi-cell phytoliths and the annotator tool for single-cells. Unprocessed and processed image tiles can be queued in the sorting application and annotated on an independent schedule by the trained annotation team. These pause points allow the imaging and annotation to proceed in parallel further increasing overall pipeline throughput.

**Table 2:** Workflow timing table.

Step	Procedure	Estimated time	Notes
1	Plant part sorting and preparation	Variable	Allow 20min per species; scales with number of individuals and anatomical parts sampled
2	Dry ashing in kiln	3.5 hours	Includes combustion; duration varies by taxon and tissue density
3	Ash homogenisation and slide mounting	3 slides 2-4min	Includes transfer to vials, slide preparation, and mounting medium application
4	Slide drying	Min 2 weeks	Pause point 1. Slides must dry horizontally; do not proceed to imaging until fully cured
5	Nikon AI annotation and model training	48 hours over weeks	Includes manual segmentation of 10× scan tiles and 1,000 training iterations; one-time step per morphotype class; does not need to be repeated for subsequent slides of the same class
6	Full-slide scan at 10× (automated JOB)	~2 hours and 40 min per slide	Includes autofocus routine and Z-stack acquisition across full slide area
7	AI-based region of interest detection (GA3)	40 minutes per slide	Runs automatically at end of 10× scan within the JOB sequence
8	High-magnification Z-stack acquisition at 40×	~1 minute per point; ~5 hours for 300 points	Runs as a continuous automated sequence requiring no operator input. Variable depending on point density.
9	EDF rendering	~2.2 seconds per image	Integrated into JOB; runs concurrently with acquisition
10	TIFF export	~60 milliseconds per image	Pause point 2. TIFF files can be stored and converted asynchronously; imaging can continue immediately
11	TIFF-to-JPG conversion and cloud upload	Runs concurrently with steps 6–10	Asynchronous pipeline; does not interrupt acquisition
12	Segmentation and tiling of Multi-cells	Minutes per batch	Automated; produces 36 standardised CNN-compatible tiles per image. Only completed for multi-cell images.
13	Image sorting and annotation	~600 curated images per week	Pause point 3. Tiles/images queue independently of imaging schedule
Total per slide (imaging day)		~9–10 hours	Steps 6–11 run as a single largely unattended session
Total weekly cycle (16 slides)		~-6 days imaging + ongoing annotation	Variable depending on point density, with operator loading confined to weekdays and unattended acquisition continuing overnight and weekends as needed

In total 4 slides can be mounted at a time and imaged using the automated workflow resulting in 16 slides imaged by the end of one week Monday-Friday.

A complete imaging session for a single slide requires approximately 2 hours and 40 minutes plus 1min per point of high magnification automated acquisition, with operator input limited to slide loading, JOB initiation, folder setup for ND2 to TIFF conversion and upload, preprocessing initialization and post-acquisition quality review totaling less than 2min. The 10× full-slide scan and POI selection accounts for approximately three hours, followed by five hours for high magnification 40× Z-stack acquisition across ~300 detected points at approximately one minute per point (points in this context were large multi-cells from grass husks). Total number of detected points will vary by slide ranging from 200–835 points (*the former being the current max number taken from a single slide, not the hardware ceiling*). EDF rendering and TIFF export proceed with the JOB sequence and do not add meaningfully to total session time on the upgraded workstation, completing at approximately 2.2 seconds and 60 milliseconds per image respectively. TIFF to JPG conversion and cloud upload run asynchronously and can proceed concurrently with subsequent imaging sessions, effectively eliminating post-processing as a bottleneck in the pipeline.

The implementation of the upgraded workstation decreased the overall time required for imaging. **Table 3A** summarizes processing speed benchmarks for the original workstation (already fitted with SSD storage at the time of benchmarking) and the fully upgraded workstation configuration. While the intermediate addition of SSD storage improved performance, CPU and memory utilization during ND acquisition, EDF rendering and TIFF export remained high (**Table 3B**), indicating that processor and memory capacity, not storage alone, remained limiting factors. Following the full system rebuild (Sect 2.3), CPU utilization for the same operations dropped to 2-22% and memory usage to 17-33/128GB (**Table 3B**) alongside the reported improvements in EDF rendering and TIFF export speed (**Table 3A**). These results indicate that sustained high-throughput acquisition depends on the combined capacity of storage, processor, and memory rather than storage alone. Users operating on HDD-based workstations should anticipate substantially longer processing times and while SSD storage is a necessary component for sustained high-throughput acquisition, our results indicate it is not sufficient on its own without adequate processor and memory capacity.

**Table 3a:** Hardware performance comparison between the original workstation (HP Z4 G5, post-SSD upgrade) and upgraded workstation configurations across key workflow operations. (sample size =180 images).

Operation	Original workstation	Upgraded workstation	Improvement
Saving as EDF	1 image per 10 seconds	1 image per 2.22 seconds	350% faster
Saving as TIFF	1 image per 33.33 seconds	1 image per 60 milliseconds	55,450% faster

Both configurations used SSD storage. Reported improvements reflect differences in CPU, GPU, RAM and system architecture rather than storage medium.

**Table 3b:** CPU and memory utilization comparison. (sample size =180 images).

Operation	Sample size	CPU utilization		RAM usage	
		Original	Upgraded	Original	Upgraded
ND Acquisition	243	17-25%	2-4%	53/128 GB	20/128 GB
Saving as EDF	187	43-60%	10-22%	45/128 GB	17/128 GB
Saving as TIFF	180	37-55%	7-9%	63/128 GB	33/128 GB

“Original workstation” and “Upgraded workstation” configurations are as defined in **Table 3A**. CPU utilization is reported as the observed range during each operation. RAM usage is reported as gigabytes used out of 128GB total system memory. ND acquisition was not independently timed per image (see **Table 3A**) and is reported here for utilization comparison only.

### 4.3 Expected training dataset yield

Training image yield varies substantially between taxa and should not be interpreted as a reflection of imaging quality or workflow failure. **Table 4** reports the imaged totals, sorted totals, phytolith training image counts, tile yield, and tiles per photo for all 34 taxa processed in this study. Tile yield, defined as the proportion of sorted tiles retained as training-quality phytolith images following curation, ranged from 0.5% (*Juncus bufonius*) to 60.4% (*Aegilops cylindrica*), with a median of 13.7% and a dataset wide mean of 13.6% across taxa (**Table 4**). On average, each imaged multi-cell photograph yielded 4.89 usable phytolith training tiles.

**Table 4:** Multi-cell phytolith training image dataset by taxon.

Taxon	Accessions	Imaged total	Sorted total	Phytolith training images	Tile yield % (Phytolith ÷ Sorted)	Tiles per photo (Phytolith ÷ Imaged)
*Aegilops*						
*Aegilops cylindrica*	1	117	4,212	2,544	60.4%	21.74
*Aegilops searsii*	2	184	6,624	649	9.8%	3.53
*Aegilops speltoides*	2	380	13,680	1,169	8.6%	3.08
*Avena*						
*Avena abyssinica*	1	172	6,192	1,270	20.5%	7.38
*Avena byzantina*	1	532	19,152	2,995	15.6%	5.63
*Avena fatua*	1	414	14,904	2,275	15.3%	5.50
*Avena ludoviciana*	1	429	15,444	334	2.2%	0.78
*Avena sativa*	3	740	26,640	2,079	7.8%	2.81
*Avena sterilis*	3	851	30,636	4,673	15.3%	5.49
*Avena strigosa*	1	545	19,620	1,384	7.1%	2.54
*Avena tartaraca*	2	605	21,780	1,738	8.0%	2.87
*Brachypodium*						
*Brachypodium distachyon culm*	1	151	5,436	unannotated	—	—
*Bromus*						
*Bromus glaucels* (culm)	1	287	10,332	1,785	17.3%	6.22
*Bromus scoparius* (culm)	1	69	2,484	unannotated	—	—
*Bromus* sp.	1	238	8,568	1,020	11.9%	4.29
*Bromus sterilis*	1	189	6,804	233	3.4%	1.23
*Festuca*						
*Festuca arundinacea*	1	188	6,768	1,825	27.0%	9.71
*Hordeum*						
*Hordeum bulbosum*	1	269	9,684	946	9.8%	3.52
*Hordeum murinum*	1	154	5,544	557	10.1%	3.62
*Hordeum sativum*	2	285	10,260	2,176	21.2%	7.64
*Hordeum spontaneum*	3	407	14,652	4,502	30.7%	11.06
*Hordeum vulgare*	1	219	7,884	1,079	13.7%	4.93
*Juncus*						
*Juncus bufonius*	1	110	3,960	20	0.5%	0.18
*Lolium*						
*Lolium persicum*	1	266	9,576	3,288	34.3%	12.36
*Lolium rigidum*	1	309	11,124	2,442	21.9%	7.90
*Stipa*						
*Stipa holosericea*	1	179	6,444	795	12.3%	4.44
*Triticum*						
*Triticum aestivum*	5	454	16,344	952	5.8%	2.10
*Triticum boeoticum*	3	618	22,248	1,429	6.4%	2.31
*Triticum dicoccoides*	3	548	19,728	3,057	15.5%	5.58
*Triticum dicoccoides* (culm)	1	100	3,600	2,095	58.2%	20.95
*Triticum dicoccum*	3	555	19,980	518	2.6%	0.93
*Triticum durum*	1	129	4,644	550	11.8%	4.26
*Triticum monococcum*	2	497	17,892	3,472	19.4%	6.99
*Triticum stilpa*	1	38	1,368	354	25.9%	9.32
*Triticum turanicum*	1	157	5,652	1,491	26.4%	9.50
Total	56	11,385	409,860	55,696	13.6%	4.89

All images refer to multi-cell images. Image totals reflect both automated and manual image acquisition. Unless designated imaged plant part is husk. Values reported here reflect raw imaging and curation output per taxon prior to any class-balancing step. Accessions column indicates the number of distinct individual plants sampled per taxon. Brachypodium distachyon (culm) and Bromus scoparius (culm) completed the full imaging and segmentation pipeline but have not yet been reviewed by a human annotator at the time of writing; both are included in the reported taxon and accession totals.

Accessions imaged, sorted, and converted to training data across 34 grass and rush taxa.

This variability is governed primarily by taxon specific morphological characteristics rather than by imaging performance. Taxa producing larger, morphometrically stable, and visually distinct multi-cell husk phytoliths, such as *Aegilops cylindrica*, yield higher tile yields during annotation. Taxa with smaller phytoliths, greater morphological overlap with background debris/artifacts, or lower phytolith density on the slide, such as *Juncus bufonius*, produce lower tile yields. The lower tile yields seen in *Juncus bufonius* may also reflect the lack of NIKON AI training on non grass phytoliths. Users should therefore anticipate that the imaging effort required to achieve balanced class representation will vary substantially between taxa and should plan acquisition schedules accordingly. Tile yield and tiles per photo data of the kind reported in **Table 4** can be used to model the number of slides required per taxa to reach a target training image count.

### 4.4 Challenges and troubleshooting

Challenge 1 – Blurred Z-stacks due to focal plan inheritance from 10x scan. Z-stacks generated using the Z-coordinate inherited from the 10× overview scan will frequently fall outside the optimal focal plane at 40×, due to slide unevenness, mounting medium thickness variation, and microtopography beneath the coverslip. A significant proportion of captures may be entirely blurred if per-coordinate autofocus is not implemented. *Resolution*: ensure that a continuous in-range autofocus routine is configured to execute prior to each 40× Z-stack within the JOB sequence. This dynamically re-centers the focal origin at every coordinate and is non-optional for consistent image quality.

Challenge 2 – NIS-Elements instability and crashes during batch TIFF export. Without the *Keep Selected Frames* function enabled, TIFF and EDF files will retain embedded metadata from all coordinates in the acquisition session, producing files of 688 MB or more per image and ND2 container files of approximately 40 GB. At this scale, batch export operations will saturate local storage, cause NIS-Elements to stall or crash, and freeze the workstation. *Resolution:* enable *Keep Selected Frames* prior to EDF rendering and TIFF export. This reduces each output file to a self-contained unit containing only the data for that imaging point, dramatically reducing file sizes while preserving image quality.

Challenge 3 – Acquisition density ceiling on HDD-based workstations. Users operating on HDD-based workstations should anticipate an effective ceiling of approximately 40 points per slide before system instability, prolonged write times, or software crashes occur. This ceiling is governed by storage I/O throughput rather than by optical or software limitations. *Resolution:* relocate the NIS-Elements job database and output directory to SSD storage as a minimum intervention. Transitioning to a fully SSD-based workstation with sufficient RAM raises the reliable acquisition ceiling to in excess of 800 points per acquisition.

Challenge 4 – AI detection selecting debris and sub-threshold fragments. In initial configurations, the NIKON AI model may flag very small phytolith fragments or multiple locations on large multi-cell phytoliths as candidate regions, increasing redundant imaging and reducing effective throughput. *Resolution:* integrate area-based object filtering by linking size filtering parameters to the GA3 detection operation, excluding objects below a defined minimum area threshold. Threshold values should be calibrated empirically for each morphotype class during the initial training phase.

Challenge 5 – Low conversion rates due to slide preparation quality. Slides with uneven ash distribution, clumping, or high background debris will produce lower phytolith density and greater annotator ambiguity, reducing conversion rates during curation. This is the upstream constraint most directly within operator control. *Resolution:* thorough plant part sorting prior to ashing and careful, even distribution of ash during mounting are the most effective interventions. Well prepared slides consistently produce higher conversion rates.

Challenge 6 – Very low conversion rates for specific taxa. Some taxa will consistently produce low conversion rates regardless of slide quality, due to inherent morphological characteristics: small phytolith size relative to the field of view, low phytolith density in the tissue type sampled or high morphological overlap with background debris. *Resolution*: if a taxon consistently yields very low conversion rates, consider whether the anatomical part being sampled is the most phytolith-productive tissue for that species, and whether adjusting sampling strategy improves yield. These taxa will require substantially more imaging time and likely more mounted material to reach the target training image counts and should be flagged during acquisition planning.

## 5 Discussion

### 5.1 Advantages of the workflow

The primary advantage of this workflow over the manual and semi-automated alternatives is throughput. With operator input confined to slide preparation, and session initiation (approximately 2 minutes per slide) compared to a minimum of 3–4 hours of continuous manual photography previously required for slides, a single researcher can sustain a weekly imaging cycle of 16 slides, generating on the low end approximately 3000 curated training images per week, without dedicating continuous working time to microscope operation. The largely unattended nature of the acquisition session is a central feature of the design, enabling a single operator to run the pipeline on multiple slides in parallel with other laboratory tasks, thereby expanding the species level breadth of training data.

A second expected advantage is improved reproducibility. By replacing manual point selection with AI-assisted detection and programmable stage control, the workflow is designed to reduce operator-to-operator variability in coordinate selection and ensures that acquisition parameters, Z-stack depth, autofocus range, EDF rendering, are applied consistently across all slides and sessions. The physical slide itself remains fixed, and point-of-interest selection is now performed by a consistently applied, algorithmically trained detection model rather than by individual operator judgement. This is an anticipated benefit of automation rather than an empirically measured outcome, as a formal comparison of inter-operator coordinate selection variability between the manual and automated workflows was not conducted in this study. The fixed 36-tile segmentation grid further standardizes image inputs to the DL model, ensuring that training data are morphologically and dimensionally comparable across taxa and annotators.

A third advantage is accessibility. The workflow described here is designed for laboratories equipped with standard research upright microscopes, infrastructure that while representing a significant investment, are widely distributed across university departments in biology, material science, geology and archaeology, that serve multiple research functions beyond phytolith imaging. This contrasts with recently published pipelines such as Sorometry (Ramón et al., 2026 preprint), which rely on dedicated digital pathology scanners such as the Olympus VS200, instruments purchased specifically for high-throughput whole-slide digitization and rarely found outside medical and specialized imaging facilities. A laboratory already equipped with a motorized research upright microscope and automated capable software such as NIS-Elements can complement the workflow described here without additional hardware acquisition, repurposing existing infrastructure for DL training data generation. The downstream components of this pipeline, including the segmentation, tiling and sorting tools impose no additional hardware requirements and can be operated on a standard laboratory workstation. For research groups whose institutions already support microscopy infrastructure for broader scientific purposes, this represents a meaningful reduction in the practical barriers to building high-quality labelled phytolith training datasets.

### 5.2 From acquisition to curation: a shifting bottleneck

These results indicate that dataset expansion is constrained not by imaging throughput or segmentation capacity, but by taxon-specific morphological clarity, phytolith size, and supervised labelling. Importantly, the observed variability in conversion efficiency does not reflect loss of images, but rather differences in diagnostic suitability. In several taxa phytoliths may appear smaller within the field of view, partially obscured, or morphologically less distinct, reducing their eligibility for inclusion in the supervised training dataset. In contrast, taxa that produce larger, morphometrically stable and visually diagnostic morphotypes yield higher retention during curation. Quantifying training image output at the curation stage therefore provides a necessary metric for estimating imaging effort required to achieve balanced class representation and for modeling future dataset scalability in supervised classification frameworks.

. The automated pipeline was designed to preserve the focus, consistency, and image quality of the previous semi-automated workflow (Section 4.2, **Table 2**), as the automated workflow uses identical optical configuration, Z-stack parameters, and EDF rendering settings to the prior manual/semi automated approach (Section 3.3.4). Image quality is a function of these fixed acquisition parameters rather than of whether capture is manually or automatically triggered. Representative output is shown in **Figure 3**. However, a key finding emerging from deployment is that the primary constraint on dataset growth has shifted from image acquisition to post-acquisition curation. Dataset growth is therefore constrained by two compounding factors: the proportion of curated images that meet the quality threshold for inclusion, and the rate at which suitable images can be reviewed and labelled by the annotation team. Even under optimized acquisition conditions, annotation capacity represents a ceiling on weekly training data output. The current workflow reliably captures in excess of 800 imaged phytoliths per slide, and the segmentation pipeline processes these into thousands of imaged tiles. However, tile yields across taxa range from 0.5% (*Juncus bufonius*) to 60.4% (*Aegilops cylindrica*) (median 13.7%, mean 13.6%; section 4.3), reflecting substantial taxon specific variation in the diagnostic suitability of captured images, a pattern driven by morphological characteristics inherent to each taxon rather than by any limitation in imaging throughput. For example, multi-cell phytoliths, in particular grass and cereal husk multi-cell forms, can be identified with higher taxonomic specificity (genus/species) using AI, however such identifications require articulated multi-cell forms that feature certain characteristics (See Rosen, 1992; Ball et al., 1996; 1999). This introduces a scale, whereby some multi-cell tiles will be more informative for the model, or ‘better’ training data than other tiles. In practical terms, this means that scaling the dataset is no longer primarily a question of microscope time or computational throughput, but of supervised labelling effort and the morphological suitability of the material being imaged.

Critically, a significant upstream constraint also operates at the level of sample preparation. The thoroughness with which plant parts are sorted prior to ashing, and the care taken during slide creation, directly determines the density and clarity of phytoliths in the final mounted preparation. Cleaner slides, those with minimal organic debris, well dispersed phytoliths, no air bubbles and consistent mounting medium coverage, produce substantially higher quality images, reduce annotator ambiguity and ultimately increase conversion rates by presenting phytoliths that are morphologically unobstructed and suitable for training. Inadequate sorting or inconsistent slide preparation therefore compounds downstream curation challenges, reducing the yield of usable training images regardless of imaging throughput. This reframing has implications beyond the present study. As automated imaging workflows become more accessible, dedicated investments toward scaling machine learning applications should increasingly focus on the curation stage, developing clearer standards for annotator training, and quality control protocols that can be reported alongside datasets to ensure reproducibility. The sorting and annotation applications developed here represents a first step towards streamlining this stage, but the results suggest that further investment in semi-automated pre-filtering could substantially improve the ratio of imaging effort to usable training data, particularly for taxa with low intrinsic conversion rates.

### 5.3 Transferability and platform independence

While the automated imaging pipeline relies on Nikon NIS-Elements for stage control, Z-stack acquisition, EDF rendering, and the AI-based phytolith detection model was trained and deployed within the NIS-Elements GA3 environment, this hardware dependency does not fundamentally limit the replicability of the workflow. The underlying imaging logic, that being full slide scanning at low magnification, AI-assisted identification of regions of interest, followed by targeted high magnification Z-stack capture with EDF rendering at each coordinate, is conceptually platform agnostic and reflects standard best practices in automated microscopy. Researchers working with other systems would follow the same sequential logic using equivalent automation modules available in other major microscopy platforms, adapting stage control, autofocus routines, and detection model training to their local software environment. The downstream components of the pipeline are entirely independent of the acquisition platform: the TIFF to JPG conversion application, the segmentation and tiling workflow adapted by Berganzo-Besga et al. (2022), the custom image sorting application and the annotation tool all operate on standard image files and can receive input from any imaging system capable of exporting high resolution brightfield z-stack composites (computer uploading, processing and sorting applications can be found at https://github.com/Ramsey-Environmental-Archaeology-Lab/Automating-the-Bottleneck.git). The workflow described here should therefore be understood as a transferable methodological framework rather than a Nikon-specific protocol.

### 5.4 Regional scope and relationship to existing pipelines

The imaging and curation workflow was developed in direct service of an ongoing DL research project which aims to create a Southern Levant phytolith AI identification workflow. At present, the dataset is being used to train classification models targeting two related problems: the possible discrimination of domesticated versus wild wheat based on multi-cell phytolith husk morphology, and species level identification across the regional range of grass husk phytoliths. These represent tasks of direct archaeological relevance, as the ability to distinguish domesticated *Triticum sp*. from wild progenitors or other wild grasses in archaeological assemblages would substantially advance our understanding of pre-domestic cultivation, early domesticatory relationships and ultimately the advent of agriculture.

The taxonomic scope of the present dataset is also worth situating explicitly within the workflow structure and the broader landscape of available training resources. Sorometry (Ramón et al., 2026 preprint), the most comprehensive phytolith AI pipeline available to date, was developed and validated on assemblages from the Bolivian Amazon, with a training set dominated by palms, sedges, maize and other Neotropical taxa, with a focus on single-cell morphotypes. The needs of the workflow presented in this paper are tailored to a near Eastern plant assemblages it has been designed around, with a focus on optimizing image quality of both multi-cell and single-cell phytoliths. Cereal and grass husk multi-cell morphotypes are among the most archaeobotanically significant plant remains for understanding domesticatory relationships and the transition to agriculture in Southwest Asia. Accordingly, this workflow has been designed to meet the imaging needs of the regional phytolith research environment. These two pipelines are complementary rather than competing resources, together beginning to build the taxonomic diversity that generalizable phytolith AI classification will ultimately require.

### 5.5 Conclusions and future directions

This study presents a validated, high throughput imaging workflow for scalable phytolith DL applications. By integrating AI-assisted detection with automated Z-stack acquisition and standardized pre-processing the pipeline enables a single operator to generate 200–800 training images per slide across 16 slides per week, without continuous microscope supervision. A key finding is that the primary constraint has shifted from image acquisition to post-acquisition curation, reframing where future investment in scaling ML applications should be directed. Developed using widely available research microscopy infrastructure and validated on 34 taxa, the workflow is accessible to laboratories without dedicated scanning equipment, lowering the practical barriers to larger scale DL dataset generation in plant science and paleoethnobotany.

The dataset generated through this workflow is subject to a minimum threshold of 500 curated training images per class before any taxon enters the model training pipeline. Taxa currently below this threshold represent active acquisition targets rather than completed classes, and ongoing imaging cycles are prioritized accordingly using the conversion rate data in **Table 4** to estimate the number of additional slides required per taxon. This standard was established to ensure that no class is trained on insufficient data. However, the precise relationship between training image count and model accuracy requires further investigation, in particular, identifying the threshold at which accuracy begins to degrade as class size decreases and whether the optimal minimum threshold shifts as the number of classes in the model increases. These are variables that must be considered when testing and publishing DL model results.

Results from the DL models being developed using the Near East training library including classification accuracy, confusion matrices and generalization performance across taxa, will be reported in future publications. The deliberate sampling of multiple individuals per species across distinct anatomical parts maximizes intraspecific morphological coverage, reducing the risk of overfitting to a single growth condition’s phenotype. The structured curation pipeline, in which tiles are independently reviewed and classified by trained annotators, produces consistently labelled ground-truth data. Together, these design decisions reflect the quality requirements identified in prior DL phytolith literature, particularly the emphasis on dataset diversity and labelling consistency highlighted by Berganzo-Besga et al. (2022) and Díez-Pastor et al. (2024). Together, these design decisions suggest that the growing training library is well positioned to support robust model training and evaluation.

Several developments are currently underway that will substantially expand the scope and resolution of the workflow described here. In terms of taxonomic coverage, future imaging campaigns will extend beyond grass husk morphotypes to incorporate additional phytolith classes each of which will require targeted Nikon AI retraining as described above. Within the grass husk category itself, finer-grained classification targets are being developed to increase taxonomic resolution to the species level, supporting more precise archaeological interpretation of assemblage composition. On the imaging side, the workflow is being adapted to support acquisition at 100× magnification, which will capture surface detail and three-dimensional morphological features not resolvable at 40×. This higher resolution imaging is particularly relevant for species level discrimination of morphologically similar taxa where fine surface texture and ornamentation carry diagnostic information. Complementing this, a transition from Entellan to Norland 165H is currently under evaluation supported by findings from Dudgeon et al. (2026) demonstrating that the refractive index of Norland 165H produces superior image clarity, edge definition and phytolith counts relative to Entellan. In parallel, and informed by Dudgeon et al.,’s (2026) findings a modified slide preparation protocol is being developed in which ashed material is allowed to fully settle within the mounting medium (Norland) prior to setting ensuring that the majority of phytoliths are distributed across a narrow and consistent Z-range within the slide. This reduces the focal depth required during automated Z-stack acquisition, minimizes the number of focal layers needed to capture each phytolith, and improves the consistency and sharpness of EDF composites across the full slide, directly benefiting the performance of the automated imaging pipeline at scale. Together these developments will progressively increase both the breadth and the taxonomic resolution of the training dataset, moving the pipeline towards the large-scale, morphologically diverse reference library required for generalizable DL classification across the full range of phytolith types encountered within regional archaeological and paleoenvironmental contexts. As regionally tailored workflows proliferate and taxonomic coverage expands, pipelines of this kind will form the methodological foundation upon which generalizable, large scale phytolith AI classification becomes a practical reality for plant science, archaeology and paleoenvironmental research.

## Data Availability

The datasets presented in this article are not readily available because the dataset contains proprietary microscope images generated as part of an ongoing research program and includes annotated images used for AI model training and development. Due to concerns related to unpublished research materials, dataset curation, and use in ongoing model development, the full image dataset is not currently publicly available. Access to portions of the dataset may be provided by the corresponding author upon reasonable request and pending approval. Requests to access the datasets should be directed to Melanie Pugliese, melanie.pugliese@mail.utoronto.ca, https://github.com/Ramsey-Environmental-Archaeology-Lab/Automating-the-Bottleneck.git.
